# Accuracy of prescribing documentation by UK junior doctors undertaking psychiatry placements: a multi-centre observational study

**DOI:** 10.1186/s13104-019-4596-2

**Published:** 2019-09-04

**Authors:** Mrinalini Dey, Kurt Buhagiar, Farid Jabbar

**Affiliations:** 1 Institute of Ageing and Chronic Disease, University of Liverpool, Liverpool, UK; 2 Research Department, East London NHS Foundation Trust, London, UK

**Keywords:** Electronic medical records, Prescribing, Severe mental illness, Junior doctors, Psychotropic drugs

## Abstract

**Objectives:**

Medical records are critical to patient care, but often contain incomplete information. In UK hospitals, record-keeping is traditionally undertaken by junior doctors, who are increasingly completing early-career placements in psychiatry, but negative attitudes towards psychiatry may affect their performance. Little is known about the accuracy of medical records in psychiatry in general. This study aimed to evaluate the accuracy of Electronic Medical Records (EMRs) pertinent to clinical decision-making (“rationale”) for prescribing completed by junior doctors during a psychiatry placement, focusing on the differences between psychotropic vs. non-psychotropic drugs and the temporal association during their placement.

**Results:**

EMRs of 276 participants yielding 780 ward round entries were analysed, 100% of which were completed by Foundation Year or General Practice specialty training junior doctors rather than more senior clinicians. Compared with non-psychotropic drugs, documentation of prescribing rationale for psychotropic drugs was less likely (OR = 0.24, 95% CI 0.16–0.36, *p *< 0.001). The rate of rationale documentation significantly declined over time especially for psychotropic drugs (*p *< 0.001). Prescribing documentation of non-psychotropic drugs for people with mental illness is paradoxically more accurate than that of psychotropic drugs. Early-career junior doctors are therefore increasingly shaping EMRs of people receiving psychiatric care.

## Introduction

Accurate medical record-keeping is of crucial importance for patient safety and quality of care [1, 2]. While traditional paper-based records are being fast replaced by electronic medical records (EMRs), allowing easier completion and retrieval of documentation, medical records in any format have been associated with limited accuracy, predisposing to negative repercussions on patient care [1]. Psychiatric services are no exception to these pitfalls [3–5].

Historically, most routine record-keeping in UK teaching hospitals has been maintained by junior doctors, with responsibility lying across an inverse hierarchy [6]. Since 2005, a redesigned programme of post-graduate training was introduced, permitting junior doctors in their initial two “Foundation Years” (FY) or those conducting general practice specialty training (GPST) to undertake successive 4-month posts in various medical specialties, including psychiatry [7, 8]. These doctors thus work alongside other doctors undertaking specialty training as their career choice. Internationally, psychiatry has, however, been a consistently unpopular specialty among junior doctors [9] and medical students [10]. A proportion of junior doctors therefore precipitate in psychiatry placements less enthusiastically than others, which may negatively influence their performance, including that related to record-keeping. Studies have also highlighted the challenges faced by junior doctors with documenting accurate clinical reasoning for prescribing (“rationale”) [11] despite decisions being mostly made by their consultant trainers [6]. The document *Good Medical Practice* by the General Medical Council—the UK’s doctors’ regulatory body—yet sets out a duty on doctors to adhere to a defined framework for prescribing documentation [12].

Evaluating the accuracy of prescribing documentation would be an important step forward given the implications on the quality of care and safety of people with mental illness and the need to address any identified deficits. Additionally, findings may elucidate the educational needs of junior doctors undertaking non-specialist psychiatry placements, whom a priori we predicted to be largely responsible for such record-keeping [13]. We aimed to specifically: (i) evaluate the proportion of prescribing-related documentation completed by FY or GPST doctors relative to other medical grades; (ii) compare its accuracy with respect to prescription of psychotropic vs. non-psychotropic drugs, and (iii) explore correlations between documentation and time into the junior doctors’ placement in psychiatry.

## Main text

### Methods

#### Setting

The study took place within four geographically and administratively distinct acute psychiatric teaching centres with a total capacity of 193 beds in the North West of England, hosting junior doctors undertaking training in Health Education England—North West [14].

#### Data collection

Data were collected for placements between April and July 2016. 4-monthly rotations of junior doctor placements take place in August, December and April, thus aiming to evaluate record-keeping during the final placement in the training year.

The data source of this study was routine clinical documentation held in patient EMRs within the National Health Service (NHS). EMRs for all patients currently or newly admitted to these centres during the study period were accessed securely by one author (MD) in December 2016, excluding records if participants were still hospitalised at the time of data access. The following participant variables were collected: age, gender, date of admission and discharge, and primary psychiatric diagnosis on discharge. This study was intended to address preliminary questions, falling within the remit of service evaluation in line with guidance with the Health Research Authority [15] and adhering to NHS clinical governance frameworks. Collection of additional sociodemographic or clinical variables was therefore not possible given the scope of these frameworks.

For all eligible records, clinical documentation of wards rounds were searched using the EMR’s in-built search commands. Ward round documentation was envisaged to most likely contain information about drug prescribing, being traditionally led by a consultant psychiatrist. Each entry was then searched manually for documentation containing plans related to drug prescribing, extracting the following data: (i) the grade of doctor completing the documentation, (ii) whether a consultant trainer was present, (iii) the name of drug prescribed, (iv) the type of prescription: initiation, discontinuation or change in dose/formulation/time and (v) whether the rationale for prescribing had been explicitly documented, e.g. if a prescription plan was made for depression, an acceptable rationale was: “*start drug X, dose, route of administration, frequency, as patient is experiencing persistent low mood*”.

A quarter of these identified records were then randomly evaluated separately by the second author (KB) in order to ascertain inter-rater reliability of data collection aiming for 100% concordance.

#### Data analysis

Analyses were conducted using *Stata* v.14 for Windows. We calculated descriptive statistics for sample characteristics and then used Chi square tests for categorical variables and one-way ANOVA for continuous variables to compare participant characteristics across the four centres. Univariable logistic regression was used to calculate the correlation between documentation of prescribing rationale as a dependent variable and drugs dichotomised as non-psychotropics vs. psychotropics as an independent variable, computing odds ratios (OR) and 95% confidence intervals (95% CI) as risk estimates. We repeated these tests for each sub-class of psychotropic drugs, with total non-psychotropic drugs as the reference group. We also used univariable logistic regression to test the correlation between participant variables—(age, gender, LoS and diagnostic category) and our main outcome of interest. Age and LoS were dichotomised using the median of the total sample, while diagnostic category was dichotomised as severe mental illness (SMI) (schizophrenia-spectrum disorders, bipolar disorder, affective psychoses and other non-organic psychoses) and non-SMI (any other mental illness). If significant, these variables were carried forward as confounders for a multivariable logistic regression model, additionally adjusted for centre as a fixed factor. Finally, we performed extended Mantel-Haenzel Chi square test for linear trend to evaluate the relationship between documentation of prescribing rationale and time. For all analyses, two-tailed tests of significance were used and differences considered significant at an alpha level of 0.05.

### Results

#### Sample characteristics

Of the 276 patients hospitalised during the sampling time-frame, 30.0% (*n *= 80) did not have any medication changes recorded in ward round documentation, leaving 196 patients (70.0%) for onward analysis. The descriptive statistics for the total participants according to centre are summarised in Table 1. Considering only the 196 participants with documented prescribing, the sample characteristics remained uniform across all centres (age, *p *= 0.450; LoS, *p *= 0.182; SMI, *p *= 0.849), except for gender (*p *< 0.001).

**Table 1:** Characteristics of participants in the study sample according to psychiatric unit

Variable	Centre 1 (*n *= 54)	Centre 2 (*n *= 73)	Centre 3 (*n *= 63)	Centre 4 (*n *= 86)	Total sample (*n *= 276)	*P*
Documented prescribing						
*Yes* (%)	35 (65)	49 (67)	45 (71)	67 (78)	196 (71)	0.316^a^
Gender, *female* (%)^b^	20 (37)	73 (100)	0	86 (100)	178 (65)	< 0.001^a^
Age, *years*						
Mean (SD)	45 (17)	42 (15)	40 (15)	41 (14)	42 (15)	0.305^c^
Range	20–80	18–74	19–71	18–71	18–80	
Median	42	43	37	42	41	
Length of stay, *days*						
Mean (SD)	57 (71)	54 (63)	48 (65)	38 (54)	48 (63)	0.277^c^
Range	1–323	4–403	3–416	1–314	1–416	
Median	30	31	34	18	27	
Diagnosis, SMI						
*n* (%)	24 (44)	33 (45)	30 (47)	41 (48)	128 (46)	0.975^a^

*SMI* Severe mental illness, defined as schizophrenia, schizoaffective disorder, bipolar disorder and other non-organic psychoses

^a^Chi square test: documented prescribing, χ^2^ = 3.535(df = 3); gender, χ^2^ = 216.7 (df = 3); SMI, χ^2^ = 0.219 (df = 3)

^b^Only Centre 1 provides non-gender specific care

^c^One-way ANOVA: age, *F *= 1.214 (df = 3); length of stay, *F *= 1.295 (df = 3)

#### Drug prescribing

Sociodemographic variables were not associated with documentation of at least one prescription (gender, χ^2^ = 0.90, *p *= 0.343; age, χ^2^ = 3.66, *p *= 0.056; LoS, χ^2^ = 3.11, *p *= 0.078). Being diagnosed with SMI predicted significantly lower odds for having at least one documented prescription compared with people with non-SMI (OR = 0.43, 95% CI 0.25–0.74, *p *= 0.002), even in the adjusted model (OR = 0.37, 95% CI 0.21–0.66, *p*< 0.001).

The 196 participants with at least one documented prescribing generated 780 individual EMR entries, 100% of which were completed by junior doctors (FY, *n* = 704, 90.2%; GPST, *n* = 76, 9.8%) in the presence of their trainers.

Of these 780 entries, 593 (76.0%) were pertinent to psychotropic drugs. The odds for documenting the prescribing rationale for psychotropic drugs were significantly lower than for non-psychotropics (OR = 0.24, 95% CI 0.16–0.36, *p *< 0.001). None of the individual participant variables significantly predicted the likelihood of documentation of the prescribing rationale; hence the results remained unchanged in the adjusted model. Table 2 summarises the comparative analyses for various groups of psychotropic with non-psychotropic drugs. In a sensitivity analysis removing prescribing documentation completed by GPST doctors, the results of the adjusted model for prescribing rationale documentation of non-psychotropic vs. psychotropic drugs remained unaffected (OR = 0.22, 95% CI 0.14–0.38, *p *< 0.001). Additional file 1 summarises the analysed prescriptions according to type (start/stop/change), together with a breakdown of the associated rationale documentation (Additional file 1: Table S1).

**Table 2:** Documentation of prescribing rationale of psychotropic vs. non-psychotropic drugs (total *n *= 780)

Drug	Documented rationale for prescribing, yes	OR^a^	(95% CI)	*P*
	*n* (%)			
Non-psychotropic drugs	153 (81.8)	1.00	–	–
All psychotropic drugs	309 (52.1)	0.24	(0.16–0.36)	< 0.001
Antipsychotics	113 (48.7)	0.21	(0.13–0.33)	< 0.001
Anti-depressants	48 (44.9)	0.18	(0.11–0.31)	< 0.001
Benzodiazepines & hypnotic drugs	101 (55.8)	0.28	(0.17–0.45)	< 0.001
Anti-cholinergics	21 (60.0)	0.33	(0.15–0.72)	0.005
Lithium	21 (87.5)	1.70	(0.45–6.00)	0.410

*OR* Odds ratio, *CI* confidence interval

^a^Odds ratios are for unadjusted model—adjusting for confounders did not alter results

#### Time-trend of documentation

Table 3 shows the results of prescribing rationale documentation of all drugs over time. There was a statistically significant decline in the proportion of rationale documentation for any drug over the placement time-frame (χ^2^_MH_ = 19.71, *p *< 0.001). The odds for decline in rationale documentation related to psychotropic drugs was higher relative to non-psychotropics (χ^2^_MH_ = 12.09, *p *< 0.001).

**Table 3:** Time trend for documentation of prescribing rationale during the junior doctors’ 4-month placement in psychiatry

Month	All drugs		OR^a^	Rationale documented		OR^b^
	Rationale documented	Rationale *not* documented		Non-psychotropics	Psychotropics	
	*n (%)*	*n*		*n* (%)	*n* (%)	
April	133 (68.9)	60	1.00	56 (86.2)	77 (60.2)	1.00
May	181 (62.4)	109	0.75	61 (85.9)	120 (54.8)	0.83
June	81 (52.3)	74	0.49	24 (70.6)	57 (47.1)	0.55
July	67 (47.2)	75	0.40	12 (70.6)	55 (44.0)	0.50
*Total*	462 (59.2)	318	–	153 (81.8)	309 (52.1)	–

*OR* Odds ratio

^**a**^χ^2^_MH_ = 19.71, *p *< 0.001

^**b**^χ^2^_MH_ = 12.09, *p *< 0.001

## Discussion

The study has addressed two strands: (i) the accuracy of record-keeping pertinent to drug prescribing for psychiatric inpatients, hence the implications on quality of care; (ii) the related contribution by junior doctors undertaking psychiatry placements. Within UK healthcare systems, these concepts are inextricable and have not been investigated previously. We found that nearly one-third of inpatients with mental illness lacked any prescribing documentation in their ward rounds especially those with SMI. Junior doctors are considerably less likely to document the prescribing rationale for psychotropic than non-psychotropic drugs, curtailing over time as they progress in their placements.

### Quality of medical records

The  widespread absence of prescribing documentation was surprising, given that new psychopharmacological treatments are normally started for inpatients. Previous US studies amongst people with schizophrenia mirror our findings, demonstrating less accurate medical records [4] and increasingly absent prescribing documentation for those with more severe symptoms relative to people with non-SMI [5]. Inaccurate or absent documentation of treatment plans can have detrimental effects on patients, exposing them to futile future treatments or side-effects.

The inequity of health between people with mental illness and the general population is well documented [16]. Our sample, in contrast displays a more optimistic picture, with the physical health of this population attracting increased attention. This may be an inadvertent consequence of the greater familiarity with non-psychotropic drugs amongst junior doctors [17], but may also reflect the successful outcomes of guidelines aimed at improving the physical health of people with mental illness [18]. Paradoxically, early-career junior doctors may be shifting the focus towards physical health problems in people with mental illness, while unintentionally contributing to a reverse disparity of esteem between physical and mental health.

Studies evaluating medical records may potentially raise ethical issues related to patient consent to data access. However, the NHS has robust clinical governance frameworks in these instances, which were followed throughout during data collection, storage, access and analysis as per related NHS guidance [15], as potential risks have been deemed to be outweighed by benefit for patients resulting from ensuing service improvement.

### Junior doctor-related factors and training implications

All ward round documentation was completed by junior doctors highlighting the medical hierarchy in the UK [19], implying that EMRs of people with mental illness are being increasingly shaped by these doctors. Junior doctors in general, consistently perceive lack of preparedness for undertaking duties post-qualification, citing limited confidence with psychosocial concepts [20] and prescribing-related documentation [21]. The latter may be related to non-technical soft skills such as clinical reasoning and initiative, which normally attains maturity “on the job” [22]. At this stage of their training, junior doctors may therefore underestimate the implications of “prescribing rationale” and accurate documentation on patient safety. Within general hospitals, junior doctors may lack the confidence to complete accurate prescribing documentation instructed by senior colleagues, feeling uncomfortable questioning clinical decision-making [23].

High levels of stress amongst FY doctors have been identified nationwide [24] and those undertaking psychiatry placements have highlighted their uncertainty with working with patients with mental illness [25]. The combination of personal vulnerabilities and influences from mentally unwell patients, especially those with SMI, may therefore be overwhelming, affecting overall performance that declines during their placement [26].

The quality of medical records can be an indirect reflection of the quality of care, and in turn, inaccurate documentation may compromise patient safety [27]. Nevertheless, junior doctors are generally aware of patient safety and successfully reflect on safety incidents in their professional portfolios [28]. Psychiatrists are indeed ideally placed, by virtue of their training, to further nurture doctors during their earlier development [29].

### Future considerations

The benefit of using standardised templates to enhance the quality of documentation of admission to hospital has been consistently highlighted [30], hence extending their use uniformly across EMRs may enhance the quality of prescribing documentation. Since 2012, FY 1 doctors undertake mandatory induction prior to their first placement, correlated with improved performance [24]. Considerations for longer inductions at the start of each subsequent placement may be beneficial in the longer-term.

## Limitations

- Data collection was restricted to ward rounds—a formal setting potentially contributing to underperformance of junior doctors, but manual scrutiny of all other EMR entries would have been exhaustive and error-prone.
- We relied on a subjective assessment of documentation, mitigated by independent scrutiny by two authors, although there are no standardised tools for this undertaking.
- The study setting was circumscribed and the sample size relatively small, limiting generalisability. However, data were collected from four heterogonous centres with comparable participant characteristics, while participant variables were consistent with those of other inpatient services in England [31].
- The time-frame was short, albeit intentionally covering the entire final placement in the training year.
- Sub-group analysis according to junior doctor type was not conducted, given the small proportion of GPST-completed documentation.
- Evidence suggests a correlation between medical school of graduation and self-perceived performance [20], but these variables were not available for evaluation.

## Supplementary information

**Additional file 1:** Types of documented drug prescriptions: Descriptive data regarding the type of prescriptions (start/stop/change) analysed in the study. **Table S1.** Summary of the documentation of prescribing rationale according to the type of prescription.

## Data Availability

The datasets used and analysed during the current study are available from the corresponding author on reasonable request.

## Abbreviations

FY: Foundation Year
GPST: general practice specialty training
LoS: length of stay
NHS: National Health Service
OR: odds ratio
CI: confidence interval
SMI: severe mental illness

## References

[1] Mann R, Williams J (2003). Standards in medical record-keeping. Clin Med.

[2] Mathioudakis A, Rousalova I, Gagnat AA, Saad N, Hardavella G (2016). How to keep good clinical records. Breathe.

[3] Tsai J, Bond G (2008). A comparison of electronic records to paper records in mental health centers. Int J Qual Health Care.

[4] Young AS, Sullivan G, Burnam MA, Brook RH (1998). Measuring the quality of outpatient treatment for schizophrenia. Arch Gen Psychiatry.

[5] Cradock J, Young AS, Sullivan G (2001). The accuracy of medical record documentation in schizophrenia. J Behav Heal Serv Res.

[6] Ross S, Hamilton L, Ryan C, Bond C (2012). Who makes prescribing decisions in hospital inpatients? An observational study. Postgrad Med J.

[7] Health Education England. Broadening the Foundation Programme: recommendations and implementation guidance. HEE, 2014. https://www.hee.nhs.uk/our-work/better-training-better-care/broadening-foundation-programme. Accessed 6 Feb 2019.

[8] Royal College of General Practitioners. The RCGP Curriculum. RCGP, 2016. https://www.rcgp.org.uk/training-exams/training/gp-curriculum-overview/document-version.aspx. Accessed 6 Feb 2019.

[9] Goldacre MJ, Fazel S, Smith F, Labert T (2013). Choice and rejection of psychiatry as a career: surveys of UK medical graduates from 1974 to 2009. Br J Psychiatry.

[10] Ajaz A, David R, Brown D, Smuk M, Korszun A (2016). BASH: badmouthing, attitudes and stigmatisation in healthcare as experienced by medical students. BJPsych Bull.

[11] Franklin BD, Reynolds M, Shebl NA, Burnett S, Jacklin A (2011). Prescribing errors in hospital inpatients: a three-centre study of their prevalence, types and causes. Postgrad Med J.

[12] General Medical Council (2014). Good medical practice.

[13] Kelley TA, Brown J, Carney S (2013). Foundation Programme psychiatry placement and doctors’ decision to pursue a career in psychiatry. Psychiatrist.

[14] North West of England Foundation School. https://www.nwpgmd.nhs.uk/foundation-training. Accessed 6 Feb 2019.

[15] NHS Health Research Authority. http://www.hra-decisiontools.org.uk/research/. Accessed 18 Aug 2019.

[16] Hayes JF, Marston L, Walters K, King MB, Osborn D (2017). Mortality gap for people with bipolar disorder and schizophrenia: UK-based cohort study 2000-2014. Br J Psychiatry.

[17] Tully MP, Cantrill JA (2005). Insights into creation and use of prescribing documentation in the hospital medical record. J Eval Clin Pract..

[18] Royal College of Psychiatrists (2009). OP67: physical health in mental health.

[19] Tallentire VR, Smith SE, Skinner J, Cameron HS (2011). Understanding the behaviour of newly qualified doctors in acute care contexts. Med Educ.

[20] Ochsmann EB, Zier U, Drexler H, Schmid K (2011). Well prepared for work? Junior doctors’ self-assessment after medical education. BMC Med Educ.

[21] Goldacre MJ, Taylor K, Lambert TW (2010). Views of junior doctors about whether their medical school prepared them well for work: questionnaire surveys. BMC Med Educ.

[22] Higgins MP, Tully MP (2005). Hospital doctors and their schemas about appropriate prescribing. Med Educ.

[23] Jubraj B, Marvin V, Poots AJ (2015). A pilot survey of junior doctors’ attitudes and awareness around medication review: time to change our educational approach?. Eur J Hosp Pharm.

[24] Van Hamel C, Jenner LE (2015). Prepared for practice? A national survey of UK foundation doctors and their supervisors. Med Teach.

[25] Beattie S, Crampton PES, Schwarzlose C, Kumar N, Cornwall PL (2017). Junior doctor psychiatry placements in hospital and community settings: a phenomenological study. BMJ Open.

[26] Lambert TW, Turner G, Fazel S, Goldacre MJ (2006). Reasons why some UK medical graduates who initially choose psychiatry do not pursue it as a long-term career. Psychol Med.

[27] Zegers M, De Bruijne MC, Spreeuwenberg P, Wagner C, Groenewegen PP, van der Wal G (2011). Quality of patient record-keeping: an indicator of the quality of care?. BMJ Qual Saf.

[28] Ahmed M, Arora S, Carley S, Sevdalis N, Neale G (2012). Junior doctors’ reflections on patient safety. Postgrad Med J.

[29] Storer D (2002). Recruiting and retaining psychiatrists. Br J Psychiatry.

[30] Ram MB, Carpenter I, Williams J (2009). Reducing risk and improving quality of patient care in hospital: the contribution of standardized medical records. Clin Risk.

[31] Bird VJ, Giacco D, Nicaise P, Pfennig A, Lasalvia A, Welbel M (2018). In-patient treatment in functional and sectorised care: Patient satisfaction and length of stay. Br J Psychiatry.

