# Supplementary material for: Accuracy of prescribing documentation by UK junior doctors undertaking psychiatry placements: a multi-centre observational study
Source: BMC Res Notes. 2019 Sep 4;12:558. doi: 10.1186/s13104-019-4596-2 (PMC6727574; doi:10.1186/s13104-019-4596-2)
Supplement: Supplementary file 1 — Additional file 1: Types of documented drug prescriptions: Descriptive data regarding the type of prescriptions (start/stop/change) analysed in the study. Table S1. Summary of the documentation of prescribing rationale according to the type of prescription. [file 13104_2019_4596_MOESM1_ESM.pdf]

Additional file 1

Types of documented drug prescriptions

Amongst the analysed drug prescriptions, drug initiation was the most commonly documented (*n*=433, 55.5%), followed by change in dose/time/formulation (*n*=229, 29.4%) and discontinuation (*n*=118). The type of prescription did not predict the likelihood of documentation of prescribing rationale for psychotropic drugs (χ^2=3.46, *p*=0.178). Analyses are summarised below.

Table S1. Type of prescription for which the prescribing rationale was documented

Drug type	Start		Stop		Dose/time/formulation change		χ^2	(*P*)
	n	(%)	*n*	(%)	*n*	(%)		
Non-psychotropics	134	(86.5)	9	(52.9)	10	(66.7)	11.00	(<0.05)
Psychotropics	155	(55.8)	46	(45.5)	108	(50.5)	3.46	(0.178)
